# Resource selection by an ectothermic predator in a dynamic thermal landscape

**DOI:** 10.1002/ece3.3440

**Published:** 2017-10-12

**Authors:** Andrew D. George, Grant M. Connette, Frank R. Thompson, John Faaborg

**Affiliations:** ^1^ Division of Biological Sciences University of Missouri Columbia MO USA; ^2^ Smithsonian Conservation Biology Institute Front Royal VA USA; ^3^ U.S.D.A. Forest Service Northern Research Station Columbia MO USA

**Keywords:** discrete choice models, edge‐effects, habitat fragmentation, LiDAR, *Pantherophis obsoletus*, resource selection, thermal ecology, western ratsnakes

## Abstract

Predicting the effects of global climate change on species interactions has remained difficult because there is a spatiotemporal mismatch between regional climate models and microclimates experienced by organisms. We evaluated resource selection in a predominant ectothermic predator using a modeling approach that permitted us to assess the importance of habitat structure and local real‐time air temperatures within the same modeling framework. We radio‐tracked 53 western ratsnakes (*Pantherophis obsoletus*) from 2010 to 2013 in central Missouri, USA, at study sites where this species has previously been linked to prey population demographics. We used Bayesian discrete choice models within an information theoretic framework to evaluate the seasonal effects of fine‐scale vegetation structure and thermal conditions on ratsnake resource selection. Ratsnake resource selection was influenced most by canopy cover, canopy cover heterogeneity, understory cover, and air temperature heterogeneity. Ratsnakes generally preferred habitats with greater canopy heterogeneity early in the active season, and greater temperature heterogeneity later in the season. This seasonal shift potentially reflects differences in resource requirements and thermoregulation behavior. Predicted patterns of space use indicate that ratsnakes preferentially selected open habitats in spring and early summer and forest–field edges throughout the active season. Our results show that downscaled temperature models can be used to enhance our understanding of animal resource selection at scales that can be addressed by managers. We suggest that conservation of snakes or their prey in a changing climate will require consideration of fine‐scale interactions between local air temperatures and habitat structure.

## 1 INTRODUCTION

Habitat fragmentation and climate change are among the greatest anthropogenic threats to global biodiversity (Bellard, Bertelsmeier, Leadley, Thuiller, & Courchamp, 2012; Haddad et al., 2015). At regional scales, effects of climate change are synergistic with habitat fragmentation, resulting in complex stressors on ecosystem processes (Opdam & Wascher, 2004). For example, changing weather patterns associated with climate warming can interact with landscape composition and predator behavior to limit productivity of prey (Cox, Thompson, Reidy, & Faaborg, 2013; Skagen & Yackel Adams, 2012). However, incorporating climate change research into conservation efforts remains challenging for at least two reasons. First, climate change often affects ecosystems by disrupting complex biotic interactions, many of which are poorly understood (Traill, Lim, Sodhi, & Bradshaw, 2010; Tylianakis, Didham, Bascompte, & Wardle, 2008). Second, there is a spatial and temporal mismatch between regional climate models and conditions experienced by organisms (Potter, Woods, & Pincebourde, 2013). Most management guidelines have therefore been limited to broad generalizations rather than responses to specific threats (Lawler, 2009; Mawdsley, O'Malley, & Ojima, 2009). Mitigating the effects of climate change on threatened ecosystems will require an understanding of how proximate climate factors affect specific biotic interactions at scales that can be addressed by natural resource managers.

**Figure 1:**
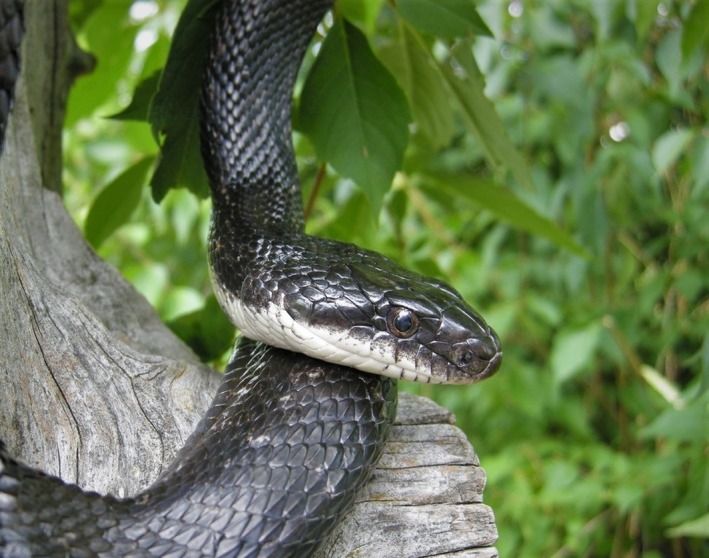
Western ratsnakes (*Pantherophis obsoletus*) are a widespread predator of birds and small mammals in eastern North America. Predation by western ratsnakes has previously been linked to global climate change and habitat fragmentation

Animal resource selection generally refers to the hierarchical process by which animals choose biotic or abiotic resources from among those that are available (Buskirk & Millspaugh, 2006; Johnson, 1980). Quantitative techniques for studying resource selection have become essential tools for understanding animal–habitat relationships and informing conservation efforts (e.g., Manly, McDonald, Thomas, McDonald, & Erickson, 2002). However, one major limitation of most resource selection studies is their assumption that resource availability remains constant through time. Traditional study designs were not equipped to account for resources, such as preferred microclimates, that can vary widely over the duration of a given study (Arthur, Manly, McDonald, & Garner, 1996; Buskirk & Millspaugh, 2006). Discrete choice modeling is a robust quantitative approach that permits resource availability to change with each animal observation (Cooper & Millspaugh, 1999). Initially developed in the social sciences, discrete choice models can provide estimates of fine‐scale selection patterns in systems where resource attributes fluctuate in time and space (e.g., Bonnot et al., 2011; McDonald, Manly, Nielson, & Diller, 2006). Here, we use discrete choice models to evaluate resource selection in western ratsnakes (*Pantherophis obsoletus*; Figure 1), a widespread predator of birds and small mammals in eastern North America.

Snakes have increasingly been recognized as regionally important predators, with the potential to exert top‐down effects on the behavior, population demographics, and evolution of prey species (Bouskila, 1995; Brodie et al., 2005; DeGregorio et al., 2014; Savidge, 1987). Because snakes are ectotherms, their behavior is constrained by ambient environmental conditions that are affected by climate change (Aubret & Shine, 2009; George, Thompson, & Faaborg, 2015a; Weatherhead, Sperry, Carfagno, & Blouin‐Demers, 2012). Recent studies have implicated snake behavior as a potential link between climate change and predation patterns, although most evidence has been indirect (Capula et al., 2016; Cox, Thompson, Reidy, & Faaborg, 2013; DeGregorio, Westervelt, Weatherhead, & Sperry, 2015). Predation by snakes has also been suggested as a possible mechanism linking habitat fragmentation to population declines in prey species, particularly birds. For example, western ratsnakes are among the most frequent bird nest predators in fragmented landscapes that likely serve as population sinks for birds (Cox, Thompson, & Faaborg, 2012; Thompson, 2007; Thompson & Burhans, 2003). Forest–field edges are hypothesized to drive fragmentation effects in some regions because fragmentation increases the ratio of edge to interior habitat (Faaborg, Brittingham, Donovan, & Blake, 1995; Robinson, Thompson, Donovan, Whitehead, & Faaborg, 1995). There is some evidence that western ratsnakes preferentially select forest‐edge habitat over forest interior, but findings have been inconsistent (Blouin‐Demers & Weatherhead, 2001; Carfagno & Weatherhead, 2006; Durner & Gates, 1993; Sperry, Cimprich, Peak, & Weatherhead, 2009).

Thermal ecology may be the most important factor affecting resource selection in temperate snakes (Reinert, 1993; Weatherhead & Madsen, 2009). Snake behavior reflects the thermal environment because virtually all aspects of ectotherm physiology are constrained by body temperature (Lillywhite, 1987; Peterson, Gibson, & Dorcas, 1993). Most snake species prefer temperatures near 30°C, although temperate species can tolerate more temperature variation than tropical species (Lillywhite, 1987). The thermal quality of a given habitat reflects the extent to which the habitat permits snakes to achieve their optimal body temperatures (Hertz, Huey, & Stevenson, 1993). Snakes must weigh thermal quality against other fitness costs, such as food availability and predation risk (Blouin‐Demers & Weatherhead, 2002; Halliday & Blouin‐Demers, 2016; Reinert, 1993). Thus, habitats that provide access to thermal heterogeneity should allow snakes to more readily thermoregulate when ambient air temperatures depart from the snakes' thermal optimum, thereby allowing more time and energy for activities such as hunting or reproduction. Locations that provide proximity to both warm and cool microclimates provide opportunities for snakes to adjust body temperatures when air temperatures become inhospitable. In contrast, thermal heterogeneity should be less important when ambient temperatures are close to the snakes' thermal optimum or during periods when energy demands are lower because the need to thermoregulate is reduced.

We conducted a large‐scale radio‐telemetry study of western ratsnakes to evaluate their resource selection in a system where they have been identified as a predominant predator of nesting birds. Previous investigations from our study sites have suggested snake behavior as a mechanistic link between songbird productivity and either climate change or habitat fragmentation. Here, our goal was to examine how the dynamic thermal landscape affects habitat preferences of individual snakes. Our use of discrete choice models allowed us to incorporate location‐specific use and availability of habitat characteristics and real‐time air temperatures within a unified modeling framework. We predicted that snakes would select habitats offering fine‐scale (<10 m) heterogeneity in vegetation structure that increases availability of optimal air temperatures, and that resource use would change seasonally to track optimal thermal conditions.

## 2 MATERIALS AND METHODS

### 2.1 Study area

We studied resource selection in western ratsnakes from 2010 to 2013 on two study sites in central Missouri. The Thomas S. Baskett Wildlife Research and Education Center (38°44′N, 92°12′W) and Three Creeks Conservation Area (38°49′N, 92°17′W) are 917 and 607 ha, respectively, and classified as Oak Woodland/Forest Hills within the Outer Ozark Border ecological subsection (Nigh & Schroeder, 2002). Cover types on the study sites included mixed oak‐hickory (*Quercus* spp., *Carya* spp.) forest interspersed with early successional red cedar (*Juniperus virginiana*) and abandoned fields. The study area has been the focus of several pioneering nest camera studies that identified predation by western ratsnakes as a major source of bird mortality in abandoned fields, near forest edges, and when air temperatures are warm (Cox, Thompson, & Reidy, 2013; Cox et al., 2012; Thompson & Burhans, 2003).

### 2.2 Radio‐telemetry

We captured snakes opportunistically by hand or using funnel traps and drift fences placed around hibernacula during spring emergence. Captured snakes were transported to a surgical facility where radio transmitters were surgically implanted using standard methods (Blouin‐Demers, Weatherhead, Shilton, Parent, & Brown, 2000; George et al., 2015a; Reinert & Cundall, 1982). We used four different transmitter models [Advanced Telemetry Systems (ATS) models R1530, R1535, R1680, R1655] ranging in weight from 1.2 to 14 g to maximize battery life in a variety of snake body sizes. Transmitters were always <3% of the snake's body mass. We monitored snakes for three days in captivity following surgery and then released them at their capture locations. All methods were approved by the University of Missouri Animal Care and Use Committee (Protocol #6605).

We tracked snakes using handheld receivers and antennas (ATS models R410, R2000, 13562, 13863) during the morning, afternoon, evening, and after dark, up to five times per week from April through September, 2010–2013. We began tracking at least 3 days after snakes were released. Individual snakes were never tracked more than once in an 18‐hr period, and the average step duration was 54.5 hr. Snakes were tracked to within 1 m of their actual locations or to the trees they were using. We recorded UTM coordinates (GPS error <10 m), and whether the snake was in a new location, the same location as the previous location, or had returned to a previously used location. Additional details regarding transmitter implantation and tracking protocols are described in George et al. (2015a).

### 2.3 Resource models

To characterize habitat structure, we used spatial layers derived from airborne light detection and ranging (LiDAR) data collected in 2009 via fixed‐wing aircraft and publically available via the Missouri Spatial Data Information Service (http://www.msdis.missouri.edu). We used the most current data available at the time of our analysis. LiDAR is a remote sensing technology that permits fine‐scale, three‐dimensional characterization of terrain, and vegetation structure across broad spatial extents (van Leeuwen & Nieuwenhuis, 2010; Vierling, Vierling, Gould, Martinuzzi, & Clawges, 2008). Program FUSION was used to generate 10‐m resolution habitat rasters from LiDAR point clouds (McGaughey, 2014). Separate rasters were created to represent forest canopy cover and understory cover, which described vegetation density higher than 3 m, and between 1 and 3 m, respectively.

We estimated real‐time, location‐specific, air temperatures using high‐resolution, spatiotemporal temperature models (George, Thompson, & Faaborg, 2015b). Temperature models were developed within an information theoretic approach, and included seasonal and hourly effects of solar radiation, vegetation structure, elevation, and weather conditions on near‐surface air temperatures. Models were fit using >120,000 temperature measurements collected from a grid of 100 remote temperature loggers (iButton^®^ model DS1021G) established across the study area, and from a centrally located weather station. Validation procedures indicated that models predicted air temperatures at 1 m above ground to within 0.01°C with high accuracy (*k*‐fold correlation coefficient = 0.98). The temperature logger array, variable selection, model‐fitting procedures, and model validation are described in detail in George et al. (2015b).

### 2.4 Discrete choice models

We performed Bayesian analysis of discrete choice models to evaluate resource selection in western ratsnakes (Cooper & Millspaugh, 1999; Thomas, Johnson, & Griffith, 2006). Discrete choice models provide an advantage over traditional measures of habitat selection because they link resource availability to specific animal locations. Thus, they enable the comparison of environmental variables that change in availability through space or time. Discrete choice models estimate the probability of an animal selecting a particular location from a choice set of potentially available locations based on its “utility,” which is a function of location‐specific attributes. We assume that each individual *j* confronts a choice among a set of alternative locations, i=1,…,I. The utility $U_{ij}$ of each alternative *i* for the given individual is expressed by

$$U_{ij}={\beta_{j}}^{\prime }x_{ij}$$

where $x_{ij}$ is a vector of measurable attributes related to alternative *i* (i.e., covariates) and $\beta_{j}$ is a corresponding vector of coefficients for these covariates that are specific to individual *j*. The estimation of individual‐specific regression coefficients accommodates variation among individuals in resource selection and accounts for the nonindependence of repeated observations of each individual (Rota, Rumble, Millspaugh, Lehman, & Kesler, 2014; Thomas et al., 2006). We treated each individual‐level regression coefficient *k* in vector $\beta_{j}$ as a realization of a normal population‐level distribution such that

$$\beta_{jk}\;∼\;N\left(\mu_{k},\;\sigma_{k}^{2}\right)$$

where $\beta_{jk}$ indicates the *k*th coefficient for individual j and the population‐level parameters $\mu_{k}$ and $\sigma_{k}^{2}$ represent the mean and variance of this selection coefficient across all individuals. The relative probability of the individual selecting alternative i from the choice set is expressed by

$$\psi_{ij}=\left(\frac{\exp(U_{ij})}{\sum_{i=1}^{I}\exp\left(U_{ij}\right)}\right)$$

where *i* represents one location from the choice set of *I* alternative locations potentially available to that individual.

We fit discrete choice models using Markov chain Monte Carlo (MCMC) methods using program JAGS (Plummer, 2003), executed in R (R Core Team 2014) using the jagsUI package (Kellner, 2015). We assigned vague prior distributions to all parameters to reflect a lack of prior knowledge of parameter values. We assumed normal prior distributions, $N\left(\mu =0,\;\sigma^{2}=100\right)$, for each of the k population‐level mean parameters and uniform, $U\left(0,\;10\right)$, for the corresponding standard deviation parameters. We ran each of three Markov chains for 25,000 iterations, discarded the first 10,000 iterations of each chain as burn‐in, and retained 1 in 15 remaining samples in order to obtain a total of 3,000 draws from the joint posterior distribution.

### 2.5 Model fitting/selection

We fit discrete choice models to our telemetry data in which each observed snake location was compared with 5 locally available locations. A 5:1 ratio of available to used points follows general guidelines established for discrete choice modeling, and permitted us to balance computational efficiency with a relatively large sample size (Cooper & Millspaugh, 1999; Northrup, Hooten, Anderson, & Wittemyer, 2013). Available locations for each choice set were randomly generated within a 376 m buffer around each snake location, reflecting the mean of the maximum linear distances moved by each individual during a 24‐hr period (Buskirk & Millspaugh, 2006; Cooper & Millspaugh, 1999). We used maximum daily distance moved rather than home range area to define choice set boundaries because ratsnakes regularly traverse their home ranges in a single day, and occasionally make forays outside their normal home range, and because we do not distinguish among orders of selection in our analysis (Johnson, 1980; Ward, Sperry, & Weatherhead, 2013; George et al., 2015a). Locations used on multiple occasions were assigned new choice sets for each occasion when they were used and were retained in the analysis (De Solla, Bonduriansky, & Brooks, 1999; Kernohan, Gitzen, & Millspaugh, 2001).

We evaluated a total of seven a priori candidate models representing plausible resource selection hypotheses for western ratsnakes (Table 1). These models included the following vegetation structure and temperature covariates: canopy cover, canopy cover heterogeneity, understory cover, temperature, temperature^2^, and temperature heterogeneity. To account for GPS error, we calculated these covariates as the mean or *SD* of raster values within a 15‐m buffer of each used and available location (i.e., approximately seven raster cells per calculation). Air temperatures at both the used and available locations were estimated for the specific time of each snake observation. We also evaluated the support for seasonal changes in resource selection by considering models with interactions between day of year and all covariates except for linear and quadratic effects of temperature. Day of year was treated as a continuous variable, although we made general inferences about early and late seasonality (George et al. 2015a). We did not consider seasonal changes in temperature preference because we assumed that physiologically optimal temperatures should remain relatively constant.

**Table 1:** Candidate models describing resource selection by western ratsnakes in Missouri

Model	Model covariates
1. Null	
2. Habitat only	Canopy cover + canopy heterogeneity + understory cover
3. Temperature only	Temperature + (temperature)^2^ + temperature heterogeneity
4. Habitat + temperature	Canopy cover + canopy heterogeneity + understory cover + temperature + (temperature)^2^ + temperature heterogeneity
5. Habitat × season	Canopy cover + canopy heterogeneity + understory cover + canopy cover × day of year + canopy heterogeneity × day of year + understory cover × day of year
6. Temperature × season	Temperature + (temperature)^2^ + temperature heterogeneity + temperature × day of year + (temperature)^2^ × day of year + temperature heterogeneity × day of year
7. (Habitat + temperature) × Season	Canopy cover + canopy heterogeneity + understory cover + temperature + (temperature)^2^ + temperature heterogeneity + canopy cover × Day of year + Canopy heterogeneity × day of year + understory cover × day of Year + temperature × day of year + (temperature)^2^ × day of year + temperature heterogeneity × Day of year

Canopy cover and canopy cover heterogeneity represent vegetation structure at heights >3 m. Understory cover represents vegetation measured at heights between 1 and 3 m.

Candidate models were ranked based on the widely applicable information criterion (WAIC), which represents a Bayesian within‐sample predictive score that is asymptotically equivalent to leave‐one‐out cross‐validation (Watanabe, 2010). Similar to AIC (Akaike, 1974), WAIC estimates the predictive accuracy of a given model with bias correction to account for over‐fitting. Stronger support for a model is indicated by a lower WAIC score. We also calculated Estrella's *R* ^2^ (Estrella, 1998) as an indication of model fit for our discrete choice models (e.g., Rota et al., 2014). Values of Estrella's *R* ^2^ range from 0 (predicts at random) to 1 (perfect fit), with intermediate values of 0.25, 0.50, and 0.75 generally considered to indicate modest, strong, and very strong predictive accuracy, respectively (Estrella, 1998). Following identification of a best‐supported model based on a comparison of WAIC values, we made the post hoc decision to test for differences in habitat selection between male and female western rat snakes by refitting the top model with sex‐specific population‐level parameters.

## 3 RESULTS

We tracked 36 male and 17 female western ratsnakes from April to September across four years. Thirty‐three snakes were tracked during at least 2 years, and seven snakes were tracked during at least 3 years. Approximately 70% of snakes were initially captured opportunistically in May and June, 2010–2012; the remainder were captured at hibernacula. Home range analysis indicated that nearly every individual used forest, field, and edge habitats (George et al. unpublished manuscript). We obtained 4650 snake locations, with 86.10 ± 41.85 ($\overset{¯}{x}$ ± *SD*) locations per individual.

All discrete choice models including either temperature or habitat covariates were better‐supported than an intercept‐only model that represented random selection of locations from the available choice sets. The top‐ranked model was the full model with habitat selection predicted by vegetation structure, air temperature, and season (Table 2). This model was a moderately strong predictor of habitat selection (Estrella's *R* ^2^ = 0.43), and indicated that habitat selection was most influenced by canopy cover, canopy cover heterogeneity, understory cover, and temperature heterogeneity. For each of these variables, 95% credible intervals for either main effects or interactions with season failed to overlap zero (Table 3). Estimated effects of air temperature were highly imprecise and were sensitive to the choice of prior distribution. However, air temperature was retained in relevant candidate models because post hoc exclusion of this variable did not meaningfully influence other parameter estimates.

**Table 2:** Candidate model ranking using the widely applicable information criterion (WAIC) for western ratsnake resource selection in Missouri

Model	WAIC	Estrella's *R* ^2^
Habitat + temperature + season	14,639.6	0.43
Habitat + temperature	15,010.3	0.33
Habitat + season	15,250.3	0.29
Habitat only	15,455.7	0.23
Temperature + season	15,532.0	0.22
Temperature only	15,605.1	0.20
Null	16,391.0	0.00

Lower WAIC values indicate greater support for a model. Estrella's *R* ^2^ provides a measure of model fit ranging from 0 (predicts at random) to 1 (perfect model fit).

**Table 3:** Estimated population‐level coefficients from the best‐supported discrete choice model describing resource selection of western ratsnakes (*Pantherophis obsoletus*)

Variable	Posterior mean	Posterior *SD*	95% Credible intervals
Canopy cover	−0.21	0.09	(−0.38, −0.04)
Canopy cover × day of year	0.11	0.06	(0.00, 0.22)
Canopy cover *SD*	0.09	0.06	(−0.04, 0.21)
Canopy cover *SD* × day of year	−0.22	0.06	(−0.33, −0.11)
Understory cover	−0.20	0.06	(−0.33, −0.08)
Understory cover × day of year	0.04	0.05	(−0.06, 0.14)
Temperature	−0.10	1.32	(−2.67, 2.60)
Temperature^2^	−0.10	0.24	(−0.56, 0.36)
Temperature *SD*	0.21	0.05	(0.10, 0.31)
Temperature *SD* × day of year	0.24	0.06	(0.12, 0.37)

Population‐level coefficients represent the expected mean response to the standardized covariate (*z*‐score) across all individual snakes.

**Figure 2:**
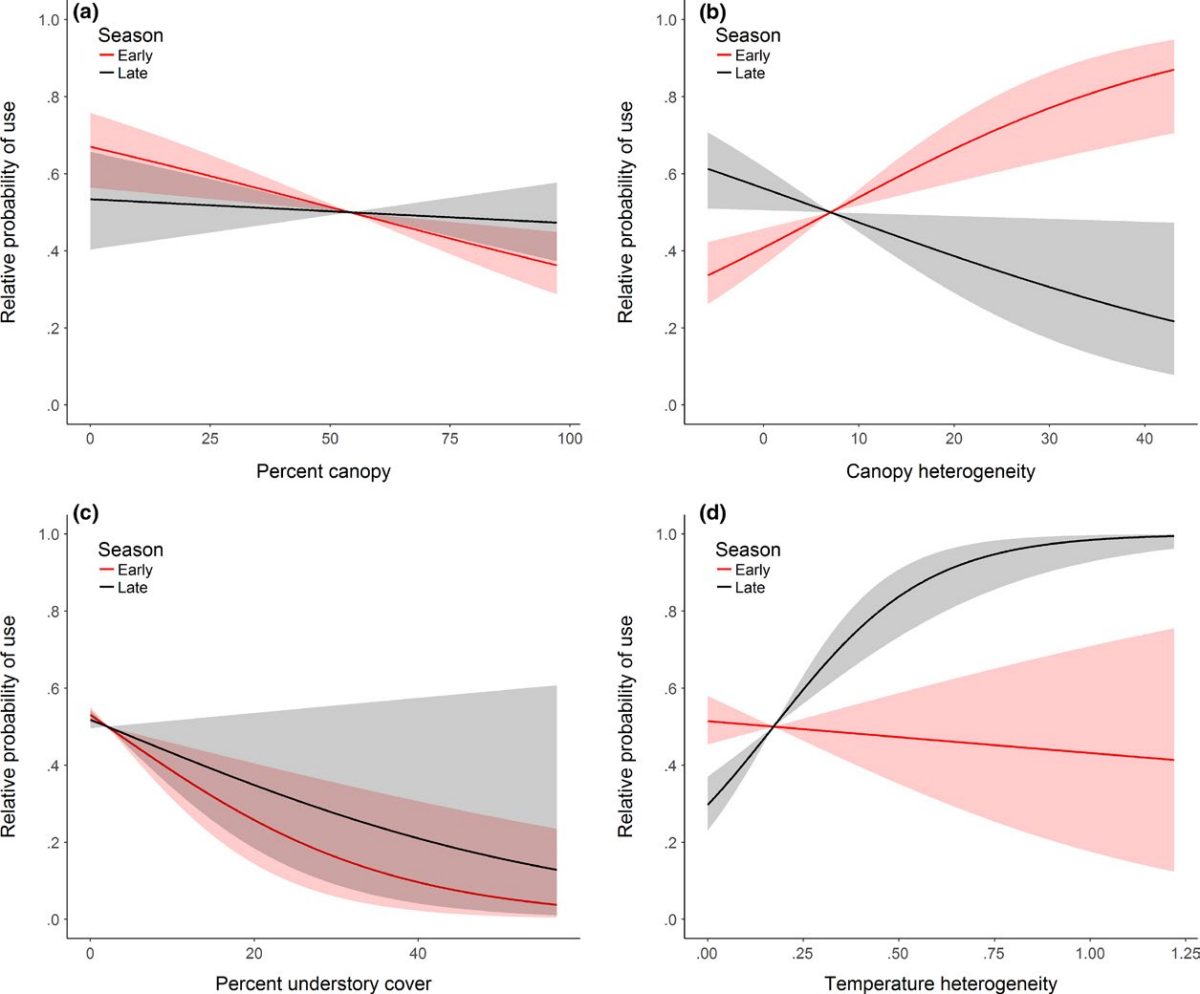
Relative probability (±95% CRI) that a location is selected by a western ratsnake (*Pantherophis obsoletus*) in relation to (a) percent canopy cover (height >3 m), (b) canopy cover heterogeneity, (c) percent understory cover (height >1 and <3 m), and (d) temperature heterogeneity. Predicted probabilities of selection are depicted relative to the observed mean value of each covariate (vertical dashed line) for both early and late in the active season (31 May vs. 31 August)

**Figure 3:**
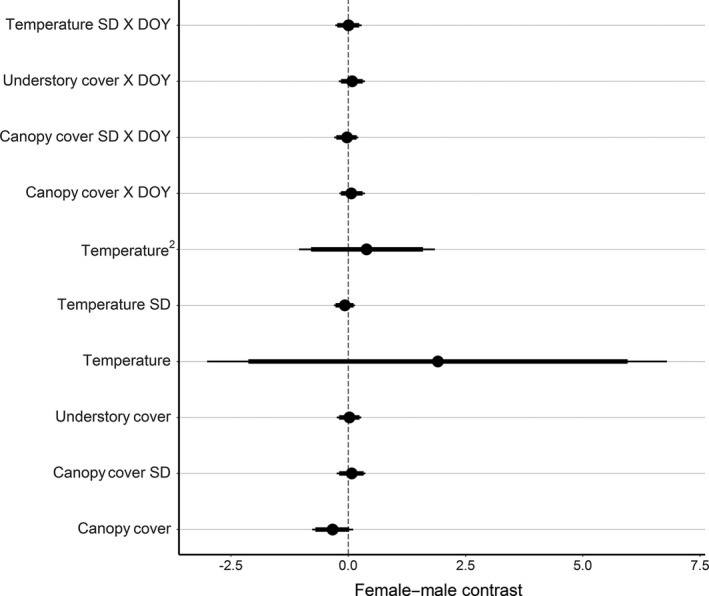
Estimated difference in standardized habitat selection coefficients between male and female western ratsnakes (*Pantherophis obsoletus*). Larger values on the *x*‐axis indicate greater preference in females than in males. Points represent posterior means, thick bars represent 90% credible intervals, and thin bars represent 95% credible intervals. The 90% credible intervals for all parameters overlapped zero

Early in the active season, individuals showed a strong preference for areas with greater canopy cover heterogeneity (Figure 2). Later in the season, individuals showed increased preference for areas with greater temperature heterogeneity. Western ratsnakes also tended to select areas of less canopy and understory cover, and there was little evidence for seasonal variation in selection for these habitat characteristics (Figure 2). Finally, refitting our top model with sex‐specific population‐level parameters provided no evidence that habitat selection coefficients differed between male and female western ratsnakes (Figure 3).

**Figure 4:**
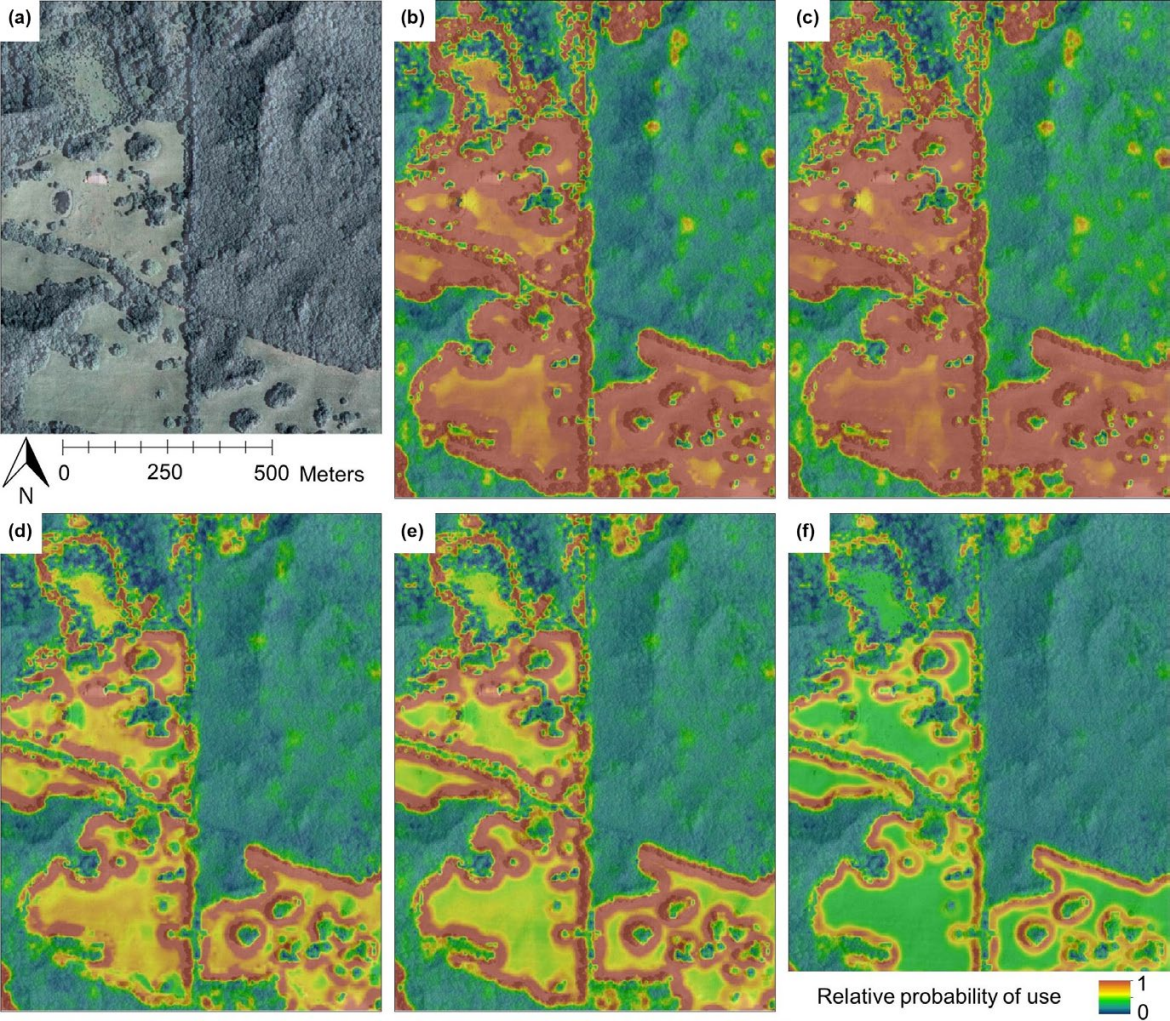
Relative probability of western ratsnake resource use at (a) a representative location on Three Creeks Conservation Area in central Missouri, USA. Panels show model predictions from the best‐supported discrete choice model for the following dates in 2013: (b) 28 April, (c) 31 May, (d) 30 June, (e) 31 July, and (f) 31 August

Spatial predictions of resource use indicated a strong positive association with forest–field edges throughout the active season (Figure 4). The best‐supported model predicted a preference for open fields and forest–field edges early in the season. Preference for open fields declined as the season progressed. Late‐season predictions indicated preference for forest–field edges over both forest interiors and fields.

## 4 DISCUSSION

Downscaling climate factors to local‐scale processes remains an important goal for forecasting and mitigating the effects of climate change (Lawler, 2009; Potter et al., 2013). We used a novel modeling framework to evaluate seasonal resource selection in western ratsnakes, a predominant predator that has previously been linked to prey population demographics (Cox et al., 2012, Cox, Thompson, Reidy, & Faaborg, 2013). We found strong evidence that western ratsnakes preferentially select habitat characteristics and thermal conditions associated with forest–field edges. Resource selection changed with day of year, shifting from preference for greater canopy heterogeneity to greater temperature heterogeneity as the season progressed. Surprisingly, mean near‐surface air temperature was not well supported as a predictor of resource selection in our models, suggesting that ambient temperatures per se may be less important than other factors affecting ratsnake habitat selection.

There is evidence from previous studies that western ratsnakes preferentially select edge habitats over forest interiors, although findings have been inconsistent (Blouin‐Demers & Weatherhead, 2001; Carfagno & Weatherhead, 2006; Durner & Gates, 1993). Forest–field edges likely provide access to optimal thermal conditions in the northern limit of ratsnakes' geographic range (Blouin‐Demers & Weatherhead, 2001). Alternatively, prey species such as rodents and nesting birds might be more abundant in edges (Fink, Thompson, & Tudor, 2006), although at least two studies did not find support for this hypothesis (Carfagno, Heske, & Weatherhead, 2006; Sperry & Weatherhead, 2009). However, prey need not be abundant in edges if adjacent habitats provide sufficient access to prey. For example, if prey abundance is high inside forests or fields relative to edges, snakes might select edges because they offer close proximity to food while simultaneously affording favorable microclimates and protection from predators. Western ratsnakes exhibit apparent familiarity to habitat features within their home ranges and commonly move >100 m per day across multiple habitat types (Durner & Gates, 1993; George et al., 2015a). Therefore, proximate food availability could affect selection of edges regardless of whether prey items are actually present in edges. The fact that nest predation rates by western ratsnakes are often higher in old fields than in forests suggests that ratsnakes may use fields near edges mainly for hunting (Thompson & Burhans, 2003).

In contrast to previous studies, our analysis made no distinctions among categorical habitat types such as edge, field, and forest. We therefore avoided making an arbitrary classification of habitat types. Model predictions identified forest–field edges as having a high probability of selection because they contain resources that ratsnakes prefer, such as structural and thermal heterogeneity. Model predictions also indicated selection for areas not traditionally classified as edges, such as small canopy gaps, or areas bordering roads and power lines. Recognition of habitat features preferred by snakes could inform management decisions regarding conservation of snakes or their prey. For example, one consequence of forest thinning or uneven‐aged management could be increased access of western ratsnakes to forest‐nesting birds (Chiavacci, Bader, & Bednarz, 2014; Cox et al., 2012).

Given the importance of thermoregulation to temperate ectotherms, it is surprising that the effect of mean air temperature on resource selection was not important in our models. However, our study design may have been poorly suited to detect temperature selection by snakes. First, temperatures in our study area typically vary more temporally than spatially. For example, alternative locations within a locally available choice set had an average range of just 0.67°, whereas temperature at a given location varied by an average of 10.16° every 24 hr (George et al., 2015b). Less than half of the snakes in our study were expected to change locations in a 36‐hr tracking interval (George et al., 2015a). Therefore, availability of air temperatures at the times snakes was tracked does not necessarily reflect availability at the specific time snakes selected locations. Second, air temperatures across the landscape reach snakes' thermal optimum (30°) almost daily throughout most of the growing season. The thermal conditions selected by snakes might be the temperature range or the amount of time within the optimal temperature range for snakes at a given time and location. Finally, the thermal quality of a habitat may not reflect actual body temperatures preferred by snakes (Hertz et al., 1993). Behavioral thermoregulation permits snakes to achieve optimal body temperature, for example, by moving between warmer or cooler microhabitats. Thus, temperature heterogeneity should be a greater determinant of a habitat's thermal quality than actual temperatures, provided that optimal body temperatures can be achieved.

The shift in preference from greater canopy heterogeneity early in the season to greater temperature heterogeneity later in the season may reflect seasonal changes in resource requirements. For example, selection of forest–field edges and small canopy openings may correspond to access to prey or breeding sites early in the season when optimal temperatures are more readily available. Proximity to temperature heterogeneity may become more important later in the summer when high daytime air temperatures and low relative humidity limit ratsnake movements (George et al., 2015a).

While ratsnakes are predominant predators in fragmented landscapes in eastern North America, their role often diminishes in forest interiors and in landscapes dominated by contiguous forest (Thompson & Burhans, 2003; Cox et al., 2012; but see Benson, Brown, & Bednarz, 2010). At least two hypotheses have been proposed to explain higher predation rates in fragmented landscapes (Chalfoun, Thompson, & Ratnaswamy, 2002). Predation rates could be higher in fragments because fragmentation inevitably places interior habitat in closer proximity to habitats that are preferred by predators (functional hypothesis). Alternatively, fragmented landscapes might support larger predator populations (numerical hypothesis), although this hypothesis has not been tested in western ratsnakes because estimating snake abundance is notoriously difficult (e.g., Dorcas & Willson, 2009). Our finding that ratsnakes preferentially select habitat characteristics and thermal conditions associated with edges provides support for the functional hypothesis, although neither hypothesis is mutually exclusive.

Global climate change is predicted to continue to affect ecosystems by disrupting biotic interactions, including interactions between snakes and their prey. However, most models that quantify species' responses to climate change suffer from spatial and temporal mismatches between climate data and the conditions experienced by individual organisms during their daily activities (Potter et al., 2013). Our results show that downscaled temperature models can be used to enhance our understanding of animal resource selection. Thus, we demonstrate an approach for bringing an understanding of climate variables within the purview of resource managers. Future work in this system should continue to investigate the link between western ratsnake behavior and prey population demographics. A logical next step will be to evaluate whether snake resource selection can predict bird nest predation patterns. Specifically, relative probability of ratsnake resource use could be included as a covariate in logistic exposure models to predict daily nest predation rates under alternate climate scenarios. Evidence linking snake resource selection to prey populations will enhance understanding of predator–prey interactions in dynamic landscapes that are increasingly subjected to climate change. Nevertheless, here we present a novel application of discrete choice models to evaluate resource selection in an important predator.

## CONFLICT OF INTEREST

None declared.

## AUTHORS' CONTRIBUTIONS

ADG and JF conceived and designed the project and oversaw data collection. GMC, ADG, and FRT developed statistical models and analyzed the data. ADG and GMC wrote the manuscript. JF and FRT provided editorial advice.
